# Prevalence and Seasonality of Influenza-like Illness in Children, Nicaragua, 2005–2007

**DOI:** 10.3201/eid1503.080238

**Published:** 2009-03

**Authors:** Aubree Gordon, Oscar Ortega, Guillermina Kuan, Arthur Reingold, Saira Saborio, Angel Balmaseda, Eva Harris

**Affiliations:** University of California, Berkeley, California, USA (A. Gordon, A. Reingold, E. Harris); Sustainable Sciences Institute, Managua, Nicaragua (O. Ortega); Ministry of Health, Managua (G. Kuan, S. Saborio, A. Balmaseda)

**Keywords:** Influenza, influenza-like illness, tropical, epidemiology, risk factors, prevalence, seasonality, research

## Abstract

Although information about seasonality and prevalence of influenza is crucial for development of effective prevention and control strategies, limited data exist on the epidemiology of influenza in tropical countries. To better understand influenza in Nicaragua, we performed a prospective 2-year cohort study of influenza-like illness (ILI) involving 4,276 children, 2–11 years of age, in Managua, during April 2005–April 2007. One peak of ILI activity occurred during 2005, in June–July; 2 peaks occurred during 2006, in June–July and November–December. The rate of ILI was 34.8/100 person-years. A household risk factor survey administered to a subset (61%) of participants identified the following risk factors: young age, asthma, and increasing person density in the household. Influenza virus circulation was confirmed during each ILI peak by laboratory testing of a subset of samples. Our findings demonstrate a high rate of ILI, with seasonal peaks, in children in Nicaragua.

## Abstract 2

Case rates were high and had marked seasonal peaks.

Influenza is a major health threat throughout the world, causing substantial illness and death each year (*1*). In temperate regions, the epidemiology and prevalence of influenza have been the topic of extensive investigation. However, data on the epidemiology of influenza in tropical countries are limited (*2*,*3*). Information concerning seasonality and prevalence is crucial for development of effective regional and global seasonal influenza prevention strategies as well as pandemic influenza control measures (*3*,*4*). This scarcity of data can be attributed to limited laboratory resources and capabilities, as well as to the lower priority given to influenza compared with other infectious diseases in these regions. Furthermore, among the small body of evidence that does exist about influenza in the tropics, insufficient data have been reported on its seasonality. Several studies have reported no defined influenza season (*5*–*7*), some have reported that influenza seasonality coincides with the rainy season (*8*–*10*), and others have reported 1 or 2 seasonal peaks of influenza activity per year (*11*–*13*).

In the absence of laboratory confirmation, potential influenza cases can be identified with a clinical definition of influenza, influenza-like illness (ILI). However, multiple respiratory viruses, including respiratory syncytial virus, parainfluenza viruses, adenovirus, and rhinovirus, can cause similar signs and symptoms (*14*). Among these viruses, only respiratory syncytial virus causes seasonal epidemic peaks in children. Because respiratory syncytial virus is most common in children <5 years of age, limiting the analysis to children >5 years of age can increase the probability that a peak in ILI is due to influenza virus (*15*,*16*). In addition, ILI has been shown to be more specific for influenza when influenza virus is circulating in an area (*17*). Finally, a more specific clinical definition can be used to increase the likelihood that ILI cases are influenza. Despite the absence of 1 commonly accepted definition for high-probability ILI, using a definition of fever >38.2°C with cough has been found to have a high positive predictive value (83%) for laboratory-confirmed influenza (*18*). To study influenza in Nicaragua, a developing country in Central America, we conducted a cohort study to evaluate the prevalence and seasonal pattern of ILI and its associated risk factors among children.

## Materials and Methods

### Study Site

Managua is the capital of Nicaragua and the largest city in the country; its estimated population is 1.4 million. The Health Center Sócrates Flores Vivas (HCSFV), a public primary care facility located in District II of Managua, served as the study site. HCSFV provides medical care for the ≈62,500 persons who reside in the surrounding catchment area.

### Study Population

We used information collected through the Nicaraguan Pediatric Dengue Cohort Study, a prospective cohort study established in August 2004 to study pediatric dengue infection. Recruitment of children 2–9 years of age was conducted by door-to-door visits in neighborhoods served by HCSFV. Children were ineligible to participate if their parents or guardians reported a history of any disease or treatment that might suppress the immune system, such as HIV/AIDS or treatment for cancer. To maintain the age structure of the cohort, during July and August of each year, additional participants 2 years of age were enrolled. Age range by the end of the 2-year study was 2–11 years. All participants were provided medical treatment and tests by study physicians at HCSFV free of charge, 24 hours per day, 365 days per year. A study ambulance was available at all times to transfer any child requiring hospitalization or emergency services not available at HCSFV. To determine the percentage of children who did not regularly attend HCSFV when ill and to encourage attendance, home visits were conducted at least 1 time per year. All children were contacted by study medical personnel each year during July or August; if the child could not be located after at least 3 attempts, the child was considered lost to follow-up.

Our analysis covered 2 years, from April 16, 2005, through April 15, 2007. As a part of the study enrollment process, study personnel used questionnaire forms that asked questions in a systematic way to gather demographic information and medical history. Subsequently, a household risk-factor questionnaire was administered at home visits and at HCSFV; children with completed household risk-factor questionnaires at the beginning or during the first few months of the time period analyzed in this study were selected as the convenience sample. The study was approved by the institutional review boards at the University of California at Berkeley and the Nicaraguan Ministry of Health. Informed consent was obtained from the parent or legal guardian of each participant, and participants >6 years of age completed assent procedures.

### Surveillance and Case Definition

Parents or guardians agreed to bring participants to HCSFV at the first sign of fever and for all medical appointments. Medical information from all visits was recorded on forms for systematic data collection at the time of consultation. The Centers for Disease Control and Prevention (CDC) definition of influenza-like illness was used: fever >37.8°C at presentation with a cough and/or sore throat. ILI episodes were considered to be the same episode if they occurred within a week of each other, if they had the same date of fever onset, or if the physician denoted them as a continuing illness. High-probability ILI was defined as a fever >38.2°C at presentation with a cough (*18*). To eliminate the younger ages in which respiratory syncytial virus, a common seasonal respiratory virus, is often prevalent, a portion of the analysis was restricted to children >6 years of age (*15*,*16*).

### Laboratory Methods

Paired serum samples were collected for a subset of participants during each peak of ILI activity (11 paired samples from the 2005 peak, 15 from each of the 2 peaks in 2006, and 10 from nonpeak periods of each year); the first sample was collected at the initial visit, and the second was collected 2–4 weeks later. The hemagglutination inhibition test was performed by using the standardized reagents and protocols provided by the World Health Organization. Results were considered positive for influenza if a >4-fold rise in hemagglutination inhibiting antibody titer was noted between acute- and convalescent-phase samples. During December 2006–January 2007, a total of 51 nasal and throat swab samples were collected and analyzed by reverse transcriptase–PCR (RT-PCR). Swabs were collected from children who had fever or a history of fever and cough and/or sore throat and symptoms for <5 days. RNA was extracted from 140 μL of viral transport medium containing the swabs by using the QIAamp Viral RNA Isolation Kit (QIAGEN, Valencia, CA, USA) and amplified by using the Access RT-PCR System (Promega, Madison, WI, USA); primers were directed to influenza A and influenza B viruses according to the standard operating procedure of CDC’s Influenza Branch.

### Statistical Methods

Total person-time was determined by the amount of time that a participant was enrolled in the cohort. For those lost to follow-up, person-time was determined by adding the known person-time between enrollment or the beginning of the study period and last contact with the study to half the amount of time between the last contact with the participant and the official loss date. Incidence was calculated as the number of ILI episodes divided by the person-time multiplied by 100. A Poisson distribution was used to calculate 95% confidence intervals (CIs) for the incidence rates. Weekly incidence was graphed and smoothed by using Lowess (*19*) with a 3-week moving average. Incidence rate ratios (IRRs) were used as the measure of relative risk. General estimating equations with a Poisson distribution were used to estimate IRRs. General estimating equations were chosen to account for the longitudinal nature of the study; most participants contributed 2 person-years of time. To check the assumption that the ILI events were uncorrelated, the models were also run with a negative binomial distribution; however, the results did not significantly differ from those of the models with a Poisson distribution, and therefore the Poisson distribution was used in all models. Robust standard errors with an exchangeable correlation structure were used to estimate the 95% CIs for IRRs. Model selection was performed by using a backward step-wise procedure. The χ^2^ test was used to compare proportions. All analysis was conducted using STATA version 9.2 (STATA Corp, College Station, TX, USA).

## Results

### Demographic Characteristics

A total of 4,276 children contributed 7,449 person-years of time to the study. Of these, 3,240 (75.8%) children contributed 2 full years of time, 555 (13%) 2-year-old children were enrolled during yearly maintenance enrollment, 118 (2.8%) children were withdrawn from the study, and 363 (8.5%) were lost to follow-up. Children were withdrawn from the study if their parents requested it, if they moved from the study area, or if they did not follow study procedures. Cohort characteristics are summarized in Table 1. Attendance at HCSFV was high; 83.3% of participants had >1 medical visit, and 94.1% of those with fever visited HCSFV by the fourth day after fever onset. At the time of home visits, only 1.9% of participants reported having sought medical care outside of the cohort.

**Table 1:** Baseline characteristics of cohort of children 2–11 years of age, Managua, Nicaragua, 2005–2007

Characteristic	All cohort participants, no. (%), n = 4,276	Participants who completed household survey, no. (%), n = 2,615	p value*
Sex			0.731
F	2,114 (49.4)	1,304 (49.9)	
M	2,162 (50.6)	1,311(50.1)	
Age, y			<0.001
2	755 (17.7)	566 (21.6)	
3	473 (11.0)	330 (12.6)	
4	539 (12.6)	348 (13.3)	
5	504 (11.8)	317 (12.1)	
6	453 (10.6)	253 (9.7)	
7	462 (10.8)	263 (10.1)	
8	460 (10.8)	227 (8.7)	
9	413 (9.7)	207 (7.9)	
10	217 (5.1)	104 (4.0)	
Asthma			0.001
Yes	259 (6.1)	213 (8.1)	
No	4,276 (94.0)	2,402 (91.8)	

*Determined by χ^2^ test.

### Incidence of ILI

**Figure 1:**
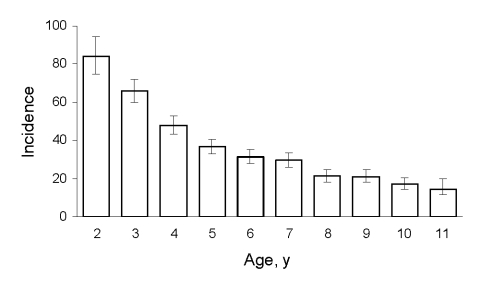
Age-stratified incidence (cases/100 person-years) of influenza-like illness in cohort of children 2–9 years of age in Nicaragua. Error bars indicate SEM.

A total of 2,596 episodes of ILI yielded an incidence rate of 34.8 episodes per 100 person-years (95% CI 33.5–36.2). Decreasing incidence was noted for each 1-year increase in age (Figure 1). Rates for boys and girls did not differ significantly (Table 2). Of the participants, 38% had had >1 episode of ILI; of those with ILI, the mean number of ILI episodes per child was 1.6 (range 1–8). Among children with ILI, the rate of hospital transfer for evaluation and possible admission was 14 per 1,000 person-years.

**Table 2:** Incidence of influenza-like illness in cohort of children 2–11 years of age, Managua, Nicaragua, 2005–2007*

Characteristic	Person-years, %†	ILI episodes‡	Incidence/100 person-years	95% CI
All participants	7,449.4	2,596	34.8	33.5–36.2
Year				
2005–2006§	3,704.5	1,106	29.9	28.1–31.7
2006–2007¶	3,745.0	1,490	39.8	37.8–41.9
Sex				
M	3,778.7	1,280	33.9	32.1–35.8
F	3,670.7	1,316	35.9	34.0–37.8
Age, y				
2	339.2	285	84.0	74.8–94.4
3	721.8	475	65.8	60.2–72.0
4	871.1	418	48.0	43.6–52.8
5	931.6	342	36.7	33.0–40.8
6	916.3	287	31.3	27.9–35.2
7	854.2	252	29.5	26.1–33.4
8	852.7	181	21.2	18.3–24.6
9	815.0	172	21.1	18.2–24.5
10	709.5	122	17.2	14.4–20.5
11	438.0	62	14.2	11.0–18.2
Asthma				
Yes	447.8	322	71.9	64.5–80.2
No	7,001.7	2,274	32.5	31.2–33.8

*ILI, influenza-like illness; CI, confidence interval. †Person-years were determined by dividing the total number of person-weeks by 52. ‡ILI was defined as an acute fever >37.8°C with cough or sore throat. §April 16, 2005–April 15, 2006. ¶April 16, 2006–April 15, 2007.

**Figure 2:**
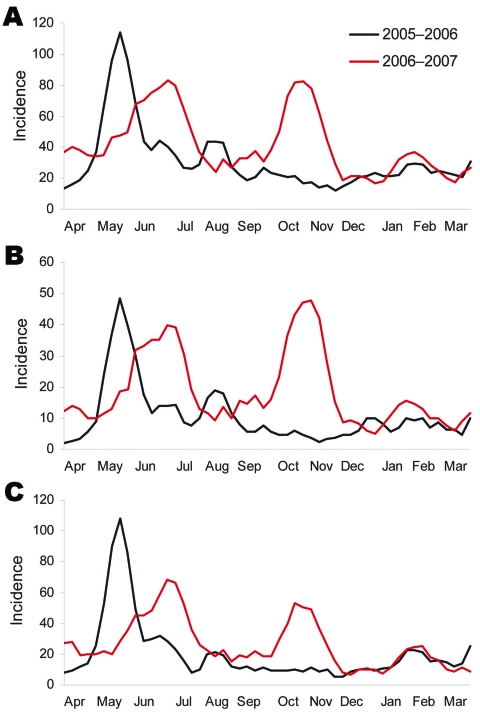
Incidence (cases/100 person-years) of influenza-like illness (ILI) in a cohort of children in Nicaragua, showing seasonal peaks, April 16, 2005–April 15, 2006, and April 16, 2006–April 15, 2007. A) Incidence of ILI episodes per calendar week. B) Incidence of high-probability ILI episodes per calendar week. C) Incidence of ILI in children 6–12 years of age per calendar week. All curves were smoothed by Lowess (*19*) by using a 3-week moving average.

ILI episodes occurred with marked seasonality; they peaked during June–July in both years and again during November–December of the second year (Figure 2, panel A). The same pattern was present when a high probability of influenza definition was used (fever >38.2°C with cough) and when the analysis was restricted to children >6 years of age (Figure 2, panels B and C). Analysis of a subset of paired serum samples under each peak showed that all 3 peaks were likely due, at least in part, to influenza; 64% of the June 2005 peak samples were positive for influenza A virus (H3N2), 27% of the June 2006 peak samples were positive for influenza B virus, and 20% of the November–December 2006 peak samples were positive for influenza A virus (H1N1) and 7% for influenza B. All samples from nonpeak times were negative for influenza A and B viruses. RT-PCR of 51 samples from the November–December 2006 peak confirmed the hemagglutination inhibition results: 10 (20%) were positive for influenza A virus. Conversely, no influenza B virus-positive samples were identified among the samples collected in June 2005, and no influenza A virus (H3N2)–positive samples were detected in November–December 2006.

### Demographic and Medical Risk Factors

Demographic and medical risk factor information was available for all cohort participants. Young age was the strongest predictor of ILI; risk decreased with each increasing year of age (Table 3). Having received a prior diagnosis of asthma was a strong predictor of ILI; risk was 1.79 times higher for children with asthma (95% CI 1.56–2.06) than for other children, after adjusting for age and sex. No significant difference in the risk for ILI was noted between boys and girls.

**Table 3:** Risk factors for influenza-like illness in cohort of 4,276 children 2–11 years of age, Managua, Nicaragua, 2005–2007*

Characteristic	Crude RR†	95% CI	Adjusted RR†‡	95% CI
Sex				
M	0.94	0.85–1.04	0.95	0.87–1.04
F	Ref		Ref	
Age, y				
2	5.94	4.49–7.85	5.53	4.19–7.32
3	4.67	3.56–6.12	4.39	3.35–5.75
4	3.46	2.63–4.54	3.29	2.51–4.32
5	2.67	2.03–3.53	2.58	1.96–3.41
6	2.30	1.74–3.03	2.24	1.70–2.95
7	2.13	1.61–2.83	2.09	1.58–2.77
8	1.52	1.12–2.04	1.49	1.11–2.01
9	1.51	1.13–2.03	1.50	1.12–2.01
10	1.23	0.91–1.67	1.23	0.91–1.66
11	Ref		Ref	
Asthma				
Yes	2.23	1.92–2.59	1.79	1.56–2.06
No	Ref		Ref	

*RR, relative risk; CI, confidence interval; Ref, reference. †The measure of RR used is the incidence rate ratio. ‡Multivariate model included sex, age, and asthma status.

### Household Risk Factors

Information concerning socioeconomic and household risk factors (hereafter referred to as household factors) was available for 2,615 (61%) participants (Table 1). The cohort and the subset of children sampled for household factors were similar with regard to sex distribution but differed slightly in age distribution and asthma status. Neighborhood geographic distribution of the entire cohort and of those with household risk factor data did not differ significantly (p = 0.98). Socioeconomic information on those surveyed is shown in Table 4. Household factors collected and included in the model were number of people living in the house, person density (determined by number of people living in the household divided by the number of rooms), mother’s literacy status, mother’s educational level, type of floor, and presence of a toilet or latrine. Electricity and running water in the house were not included in the model because >99% of the cohort had access to each (at least some of the time). The final model contained person density in the household, mother’s literacy status, type of floor, and the child’s age, asthma status, and sex (Table 5). A trend of increasing risk for ILI was noted with increasing person density in the household; homes with 3.0–4.9 persons per room had an RR of 1.07 (95% CI 0.96–1.20), and those with >5 persons per room had an RR of 1.18 (95% CI 1.04–1.34) compared with households with <3 persons per room. Having a literate mother was protective against ILI (RR 0.79; 95% CI 0.64–0.98). Although not retained in the final model, in the univariate analysis the mother’s educational level displayed a U-shaped pattern; risk decreased until completion of primary school and then increased with completion of secondary school or college.

**Table 4:** Household characteristics of participants in cohort of children 2–11 years of age, Managua, Nicaragua, 2005–2007

Characteristic	Participants with household data, no. (%), n = 2,615
Persons/room	
<3	1,254 (47.6)
3–4	917 (35.1)
>5	444 (17.0)
Mother literate	
Yes	2,436 (94.4)
No	146 (5.6)
Mother’s education level	
None	158 (6.1)
Some primary	421 (16.1)
Completed primary	387 (14.9)
Some secondary	1,045 (40.2)
Completed secondary	361 (13.9)
College	225 (8.7)
Type of floor	
Dirt	552 (21.1)
Concrete or other	2,063 (78.9)
Electricity	
Yes	2,605 (99.9)
No	3 (0.1)
Sanitation	
None	17 (0.7)
Latrine	307 (11.7)
Flushing toilet	2,291 (87.6)
Access to potable water	
Yes	2,582 (99.4)
No	16 (0.6)

**Table 5:** Household risk factors for influenza-like illness in a subset of 2,615 children 2–11 years of age, Managua, Nicaragua, 2005–2007*

Characteristic	Crude RR† (95% CI)	Adjusted RR†‡ (95% CI)
Sex		
M	0.96 (0.87–1.07)	0.98 (0.89–1.07)
F	Ref	Ref
Age, y		
2	5.01 (3.63–6.93)	4.83 (3.50–6.66)
3	4.21 (3.08–5.76)	4.07 (2.98–5.56)
4	3.13 (2.29–4.30)	3.05 (2.22–4.18)
5	2.55 (1.85–3.51)	2.51 (1.82–3.45)
6	2.03 (1.47–2.80)	2.01 (1.46–2.78)
7	2.01 (1.45–2.80)	2.00 (1.44–2.78)
8	1.50 (1.06–2.12)	1.49 (1.06–2.10)
9	1.55 (1.10–2.19)	1.54 (1.09–2.17)
10	1.25 (0.88–1.77)	1.24 (0.88–1.76)
11	Ref	Ref
Asthma		
Yes	1.83 (1.57–2.13)	1.51 (1.32–1.75)
No	Ref	Ref
Persons/room		
<3	Ref	Ref
3–4	1.04 (0.93–1.17)	1.07 (0.96–1.20)
>5	1.14 (1.00–1.31)	1.18 (1.04–1.34)
Mother literate		
Yes	0.80 (0.63–1.01)	0.79 (0.64–0.98)
No	Ref	Ref
Dirt floor		
Yes	0.95 (0.84–1.08)	0.88 (0.78–1.00)
No	Ref	Ref

*RR, relative risk; CI, confidence interval; Ref, reference. †The measure of RR used is the incidence rate ratio. ‡Multivariate model included sex, age, asthma status, person density in house, mother’s literacy, and a dirt floor.

## Discussion

We have documented a substantial amount of illness and the seasonal variation of ILI in a large cohort of children in Nicaragua, a tropical developing country. A high level of ILI (34.8 episodes/100 person-years) was found for all age groups. Incidence was highest for those 2 years of age (84.0 episodes/100 person-years) and decreased with each age increase of 1 year. Furthermore, a seasonal pattern in ILI activity was noted; a peak occurred during June–July in each of the 2 years of the study. Additionally, in the second year, a second peak of ILI was documented during November–December; thus, Nicaragua may experience 2 peaks of influenza activity in some years. The hypothesis that the observed peaks of ILI are due to influenza is supported by the seasonal pattern of high-probability ILI, ILI in children older than 6 years (who are less likely to have respiratory syncytial virus infections) (*15*), and laboratory results.

Risk factor analysis showed that young age and asthma status were strong risk factors for ILI, consistent with what has been found in other studies (*20*,*21*). Person-density in the house was positively associated with risk for ILI. Although children in households with 3.0–4.9 residents per room had an elevated risk that did not achieve statistical significance, those in households with >5 residents per room displayed a significantly increased risk for ILI. In a study of acute respiratory infection in children in Greenland, nighttime crowding (i.e., sharing a room at night) was significantly associated with the risk for acute respiratory infections, but general crowding (i.e., number of persons/room) as measured in this study, was not (*22*). Several studies in developing countries have yielded mixed results concerning the association between household crowding and acute respiratory infection, but most report no association (*23*). Our study found that maternal literacy was protective for ILI but that the mother’s education level was not significantly associated. Although not significant in the final model, the mother’s education level showed a U-shaped relationship, perhaps because of a higher likelihood that mothers with a higher education level work outside of the home, necessitating outside childcare. Certainly, this association and others need to be examined further. In particular, including details about childcare (e.g., whether the child is cared for outside the home and the type of facility) and socioeconomic factors, will be informative. We have refined the household questionnaire and will administer it on a yearly basis.

Strengths of this study include the large size of the cohort, its prospective nature, and high compliance with study procedures. In addition, the study had a high rate of follow-up; only 11.3% of the children were either withdrawn or lost to follow-up. Children who were censored from the analysis do not appear to have differed from those who completed the 2-year period (data not shown); thus, we do not believe that our results are biased due to censoring.

One major limitation is that this study used a syndromic definition for ILI because specimens to determine causative agent were not generally available; because the cohort was not initially established to study respiratory diseases, respiratory samples were not collected for most of the study period. However, influenza during all 3 peaks of ILI activity was confirmed by laboratory testing of a subset of samples. Additionally, the study relied on enhanced passive surveillance, and thus we cannot be certain that we captured all episodes of ILI in the cohort. Nonetheless, compliance with study procedures was high; 94% of children visited HCSFV by the fourth day of fever, and only 1.9% reported having sought medical attention by nonstudy medical personnel. However, some mild ILI episodes were likely not detected because participants did not seek medical attention. Because of the passive nature of the surveillance and the requirement for fever, our calculated incidence may underestimate the true incidence of ILI in the cohort. Additionally, this study covered only a 2-year time period, which limits our ability to assess seasonality. Finally, the sample of participants that participated in the household survey did differ somewhat from the general population, likely because of an increased probability of parents of younger children being home in the daytime. However, because the geographic distribution of participants included in the risk factor analysis did not differ significantly from the distribution of the general cohort and because in Nicaragua, neighborhood is strongly associated with socioeconomic status and living conditions, it is likely that the subset for whom risk factor data were available was reasonably representative of the cohort, after age and asthma status were taken into account.

Influenza, in both its epidemic and pandemic forms, is a major health threat in tropical regions, just as it is in temperate regions. The lack of data on the epidemiology of influenza in tropical regions makes it extremely difficult for nations in these areas to plan for and prevent influenza. It also hampers attempts at modeling pandemic influenza and development of appropriate control strategies. Results from this initial study of pediatric ILI in Nicaragua document a high level of disease and demonstrate pronounced seasonal peaks. Risk factors were young age, an asthma diagnosis, and high person-density in the house; a protective factor was having a literate mother.

A prospective study of influenza in the Nicaraguan pediatric cohort in which respiratory samples will be tested for influenza is currently under way. This study should further characterize the epidemiology of influenza and analyze the nucleotide sequence variation and the relationship of influenza viruses circulating in the cohort to those isolated in the Northern and Southern Hemispheres. Further studies, particularly with laboratory-confirmed outcomes, in multiple countries, are needed to confirm the seasonality and level of influenza in the tropics.
